# Molecular nature of breakdown of the folic acid under hydrothermal treatment: a combined experimental and DFT study

**DOI:** 10.1038/s41598-020-76311-y

**Published:** 2020-11-12

**Authors:** Anna M. Abramova, Alina A. Kokorina, Olga A. Sindeeva, Franck Jolibois, Pascal Puech, Gleb B. Sukhorukov, Irina Y. Goryacheva, Andrei V. Sapelkin

**Affiliations:** 1 Saratov State University, Saratov, 410012 Russia; 2 Université de Toulouse Toulouse III-Paul Sabatier, Université de Toulouse-INSA-UPS, LPCNO, CNRS UMR 5215, 135 av. Rangueil, Toulouse, France; 3 Université de Toulouse, CNRS, CEMES, 29 rue Jeanne Marvig, 31055 Toulouse, France; 4 Queen Mary University of London, Mile End Road, London, E1 4NS UK

**Keywords:** Biochemistry, Chemistry, Physics, Chemical physics

## Abstract

Using a combination of experimental Raman, FTIR, UV–VIS absorption and emission data, together with the corresponding DFT calculations we propose the mechanism of modification of the folic acid specifically under the hydrothermal treatment at 200 °C. We established that folic acid breaks down into fragments while the pteridine moiety remains intact likely evolving into 6-formylpterin with the latter responsible for the increase in fluorescence emission at 450 nm. The results suggest that hydrothermal approach can be used for production of other purpose-engineered fluorophores.

## Introduction

Folic acid (FA) is one of the most well-known and essential compounds involved in a variety of biochemical processes. As a consequence, its structure, stability and interaction with a wide range of live systems and molecular compounds under variety of conditions have been widely studied^1^. There is also particular interest in FA related to cancer cell targeting as they exhibit (compared to normal healthy cells) overexpressed folate receptors on their surface^2^. It has recently been reported that hydrothermal treatment (HT) of FA can result in the production of carbon nanoparticles generally referred to as graphene quantum dots^3^ or carbon dots^4,5^ with the average particle size from 2 to 5 nm. These new systems show increased light emission intensity while apparently retaining the cancer targeting functionality^3,4,6^. The exact mechanism of the increase in the light emission is still unclear with some papers suggesting decomposition of pterin moiety following hydrothermal treatment for 6 h at 220 °C^3^, while others suggest presence of folic acid residues following HT treatment for 6 h at 240 °C^5^. At the same time, a recent study^7^ into the mechanism of binding between the FA and folate receptors demonstrates that it is driven by a complementary charge and shape of the FA pterin moiety, thus suggesting that the shape of the pterin moiety plays the key role in the folate receptor binding. Hence, HT synthesis conditions for processing of FA should be best designed to preserve the pterin moiety if cancer-targeting functionality is desired.

The temperature stability of the FA in the solid state has been investigated previously^8^ and it was found that FA undergoes significant degradation by 200 °C. On temperature increase the total loss of glutamic acid occurs above 148 °C with loss of amide functionality by 195 °C. Further degradation occurs above 262 °C that results in breakdown of the pterin moiety. Investigation of the thermal stability of the folic acid in liquid media are less detailed, but indicate that FA is stable at temperatures below 180 °C^9^. These findings suggest that HT synthesis temperatures should typically be in arrange of up to 250 °C to avoid complete degradation of the FA.

Thus far, most of the publications on the subject of HT treatment of FA for the purpose of enhanced fluorescence report changes observed in the UV–VIS absorption signal and in the light emission in the blue range of the optical spectrum^3–6,10^. Significantly, the emission is typically found to be excitation-independent in contrast to the carbon dots prepared from other precursors^11,12^, while quantum yield reaches values of over 90%^3,5^. However, the atomic structure and the nature of the light emission in these systems remains unclear. Thus, one of the key questions about the exact structural evolution pathway of FA under the HT treatment remains unanswered, while addressing it would certainly help optimisation of HT synthesis methods for preparation of FA-based cancer-targeting fluorophores.

It has been recently demonstrated that DFT calculations can provide molecular-level information on FA breakdown under UV illumination^13,14^, suggesting this can be an affective route to establishing molecular mechanism and the nature of reaction products. We also found^15^ that a combination of optical characterisation together with gel-electrophoresis separation can provide crucial insights into the nature of the light emission in carbon nanodots. In this work we tackle the question of structural evolution of FA under HT treatment using similar methodology aided by DFT calculations.

## Results and discussion

**Figure 1:**
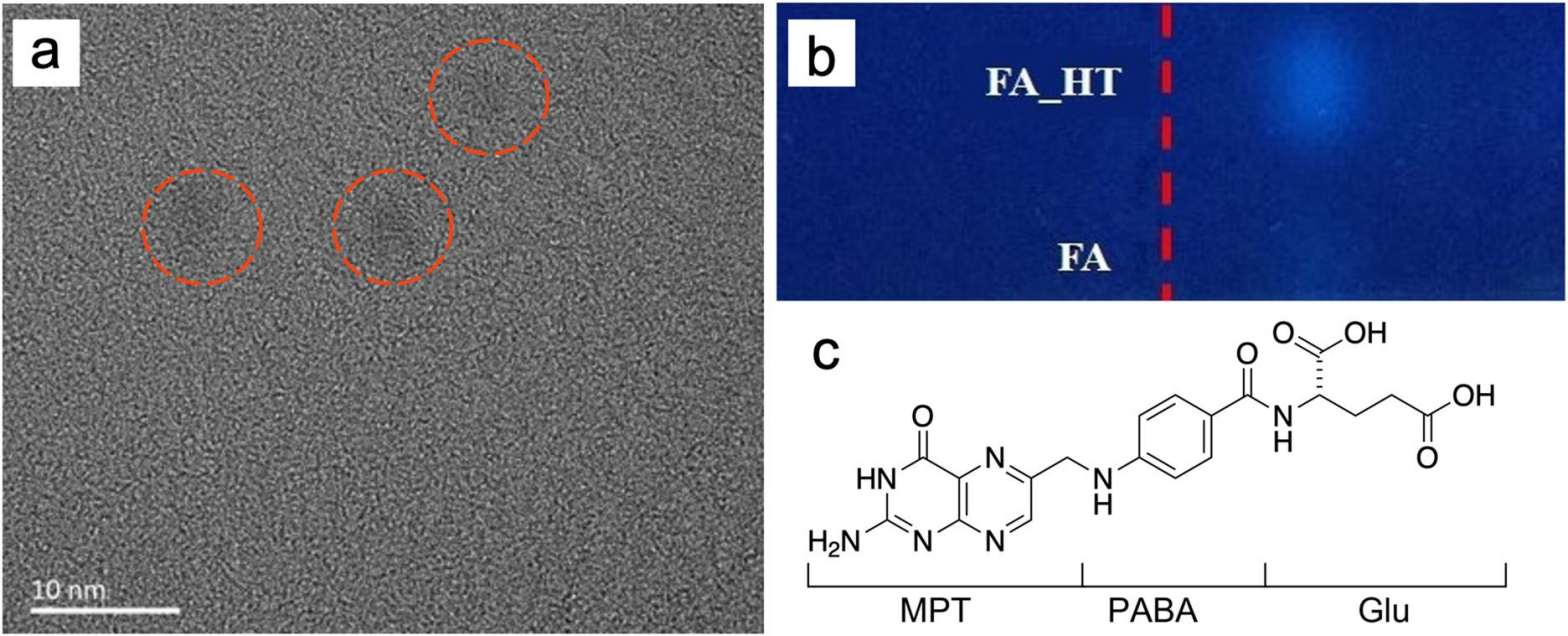
TEM image of HT FA (**a**); gel-electrophoresis photo of FA and HT-treated FA (**b**). Red dotted line indicates location of the loading wells. Main structural units of the FA (**c**).

A typical TEM image of the FA following HT treatment is shown in Fig. 1a, where one can observe several darker areas around 3–4 nm  in size. This is a typical size of the HT treated FA reported previously^3,5^ and may indicate formation of carbon nanoparticles. However, comparative gel-electrophoresis runs (see Fig. 1b and Fig. S1) clearly suggest that the size of fluorescent particles have not changed following HT treatment since their distance from the loading wells (red dashed line) is nearly identical. This suggests that the fluorophore obtained following HT treatment is likely to be molecular in nature. At the same time, the emission intensity from the HT-treated FA has clearly increased. This is supported by the quantum yield (QY) measurements in the corresponding samples (see Table S1). We also found that increasing the HT treatment time from 120 to 180 min at 200 °C results in decrease in quantum yield, hence only samples with the highest QY (9.5%) prepared over 120 min at 200 °C have been studied.

The FA molecule is composed of three moieties (see Fig. 1c): 6-methylpterin (MPT, pteridine ring), p-aminobenzoic acid (PABA) and glutamic acid (Glu). The MPT is responsible for the binding of FA by folate receptors^7^ as well as for the intrinsic light emission from the FA molecule. PABA is playing the key role in non-radioactive relaxation via the intramolecular charge transfer from the photoexcited MPT^16,17^, thus quenching the fluorescence emission. Hence, observed increase in fluorescence QY indicates disruption to the non-radiative intramolecular excitation transfer.

**Figure 2:**
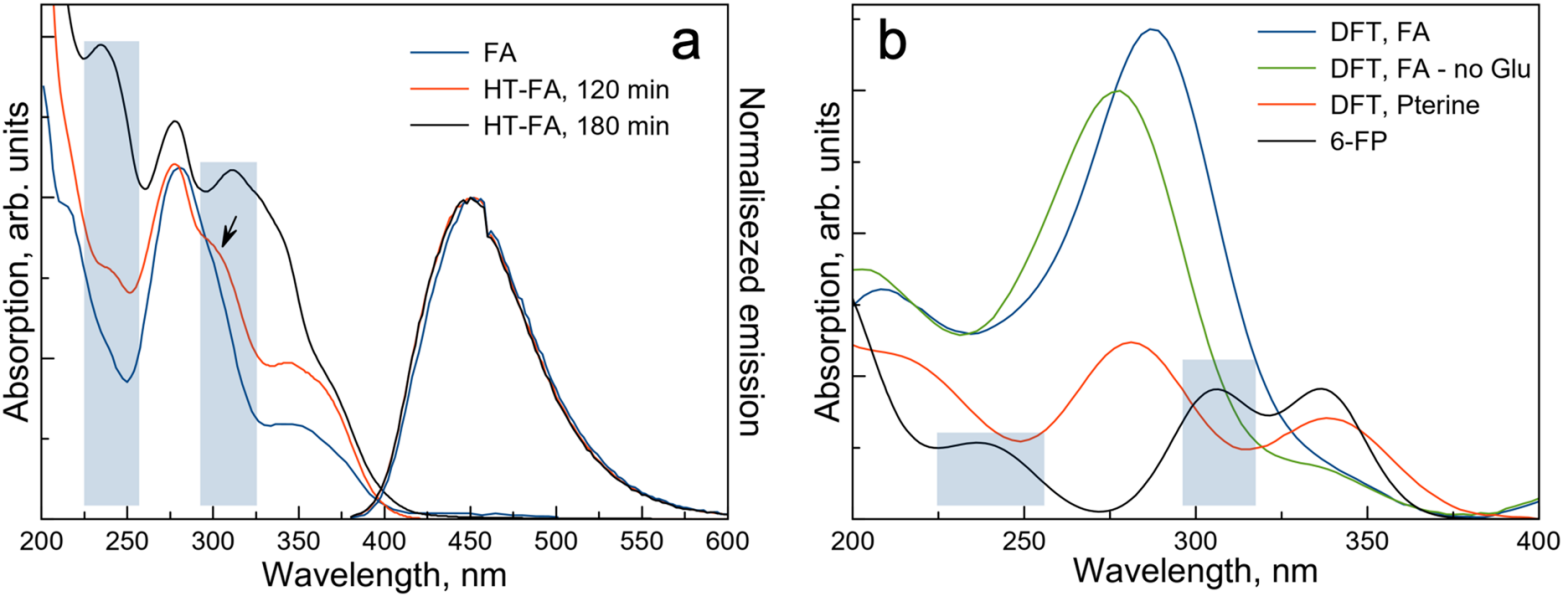
Effects of the hydrothermal treatment time on the absorption and emission spectra of hydrothermally treated folic acid solution (C = 10^–3^ M) (**a**). Emission spectra have been normalised for clarity. DFT calculations of absorption of FA: FA, FA fragment without the Glu (FA-no Glu), pterine-6-carboxilic acid (Pterine) and 6-formyl pterin (6-FP) (**b**).

Evolution of the optical absorption and of the normalised emission spectra of FA under HT treatment is shown in Fig. 2a. We found that noticeable changes (indicated by the blue shaded area) appear in the absorption signal around 120 min at 200 °C and become significant after 180 min at 200 °C. At the same time, we observed gradual increase in the emission intensity and QY (see Table S1) without noticeable changes in the emission peak shape (see Fig. 2a).

Thus, emission data suggest that the main effect of HT treatment is to disrupt the non-radiative relaxation pathway, which suggest significant PABA degradation. This is further confirmed by the DFT simulations that were used to obtain absorption signals (see Fig. 2b) for FA, FA fragment without the Glu (FA-no Glu) and finally, for two modified MPT fragments: pterine-6-carboxilic acid (Pterine) and 6-formyl pterin (6-FP). The latter two were identified as the most likely final configurations of the MPT fragment in an aqueous solution under the hypothesis of breakdown of Glu and PABA. Here we note peaks developing around 240 and 310 nm in the absorption data of Fig. 2a for 120 min sample (these are even more pronounced for 180 min sample). These peaks are also indicated by the blue shaded area in the data obtained from the DFT calculations. It is clear that these peaks correspond to the structure with no PABA (and following the dissociation of glutomate)—6-FP—indeed suggesting that the effect of HT treatment of FA for 120 min at 200 °C is the disruption of the non-radiative recombination through PABA due to FA degradation possibly to 6-FP.

**Figure 3:**
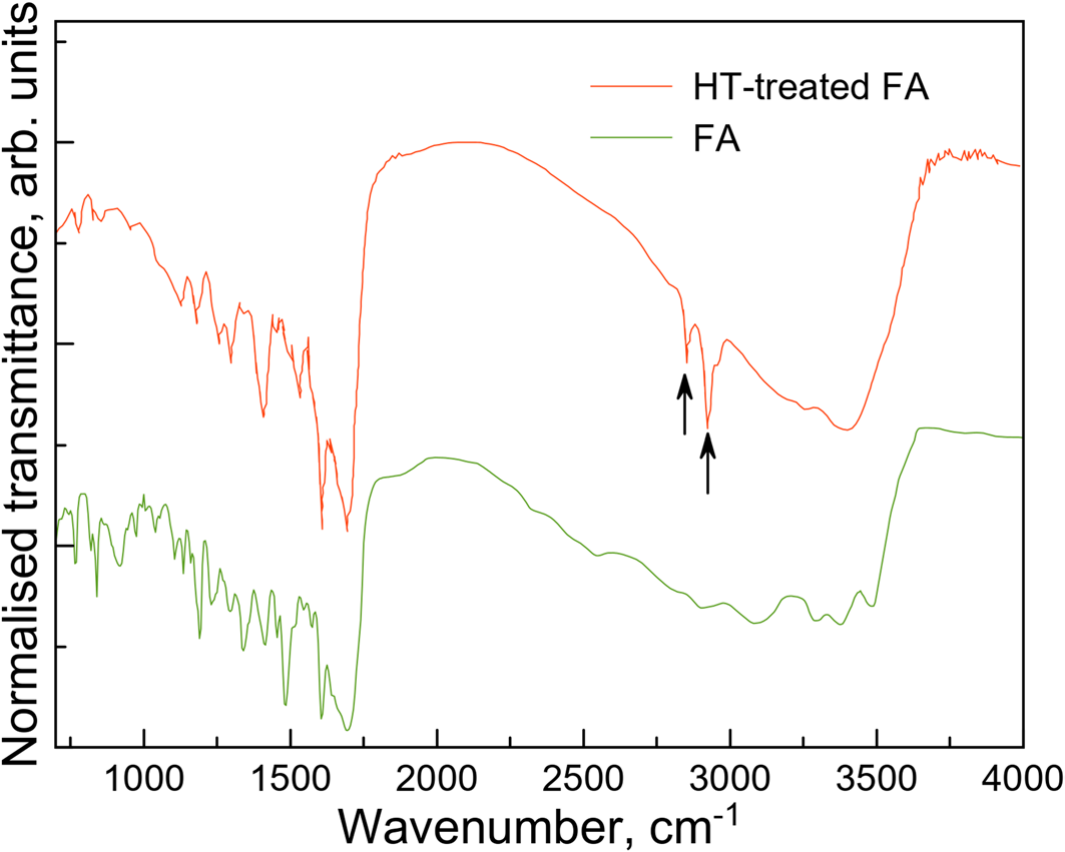
IR spectra of the FA before and after HT treatment (120 min at 200 °C).

Further insights into the evolution of the folic acid under HT treatment can be gained from the IR and Raman measurements. Infra-red (IR) spectroscopy is particularly sensitive to the hetero-nuclear polar functional group vibrations as it depends on a change in the corresponding dipole moment, while Raman is sensitive to the non-polar bonds. Hence, the combination of the two techniques can yield the overall picture of the structural evolution of the surface groups and of the molecular backbone in HT-treated FA. The most obvious changes in the IR data (see Fig. 3) are around 2820–3000 cm^−1^ where a development of strong sharp peaks can be seen (see arrows) corresponding to the C–H stretching vibrations. These are typical of alkenes^18^ possibly indicating presence of large number of products resulting from the breakdown of the Glu part of FA. Crucially, features corresponding to the presence of aromatic systems^18^ remain strong at around 1400 cm^−1^, 1500 cm^−1^ and 1600 cm^−1^, suggesting that these are largely intact. In addition, a number of broad peaks in the 2750–3500 cm^−1^ range are also retained. In the folic acid this range corresponds to the NH and NH_2_ stretching vibrations^19^ found in the secondary and primary amines, hence these seem to be largely preserved.

**Figure 4:**
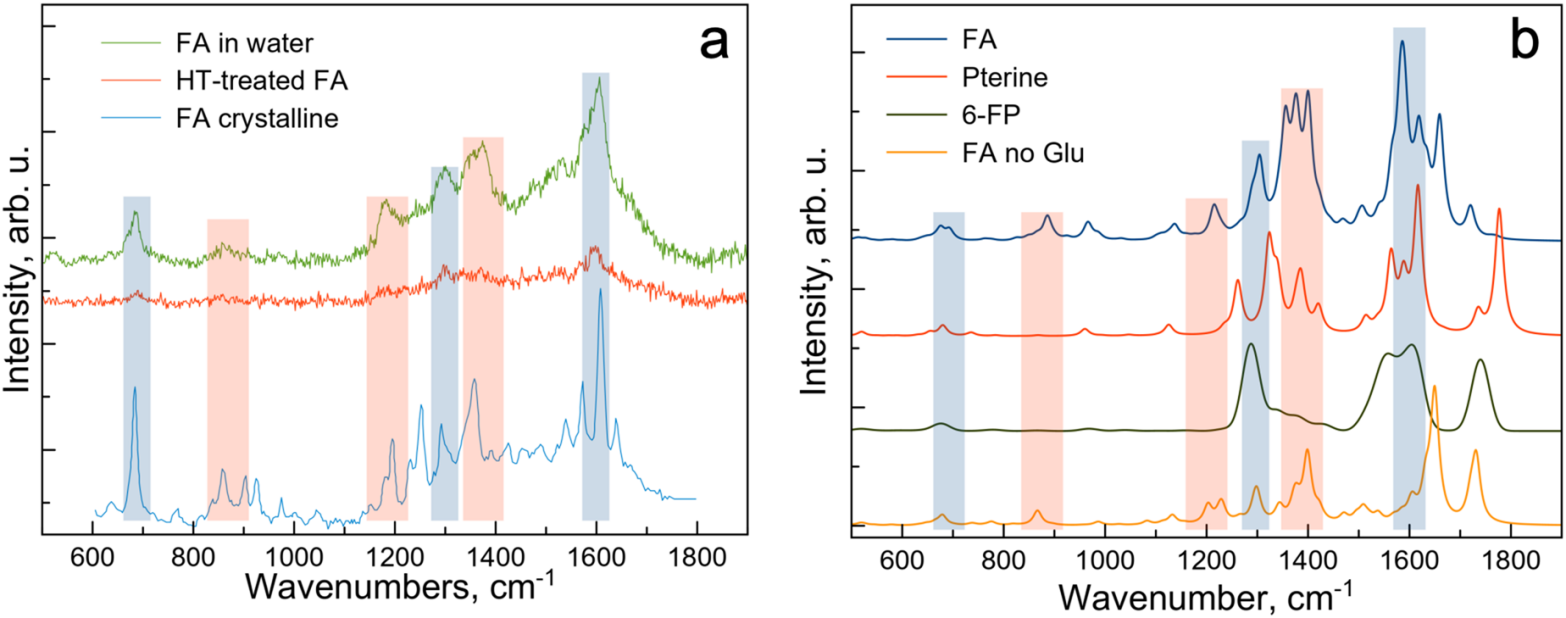
Experimental Raman data for crystalline FA, FA diluted in aqueous solution and HT-treated FA (**a**). Simulated Raman data for of FA, FA fragment without the Glu (FA no Glu), pterine-6-carboxilic acid (pterine) and 6-formyl pterin (6-FP) (**b**).

Experimental Raman data together with the spectra obtained from the DFT simulations are shown in Fig. 4. The blue shaded regions indicate areas where the Raman signal is largely preserved despite reduced signal intensity and changes in the signal shape, while in the red areas the signal is degraded. The DFT-based calculations of the Raman signal (see Fig. 4b) allow detailed assessment of the effect of FA decomposition on the Raman spectra. Comparative analysis of the Raman spectra indicate that it is peaks corresponding to the Glu and the PABA moieties that disappear as a result of HT treatment of the FA, while signals corresponding to the MPT (both in 6-FP and pterine forms) are preserved.

Thus, we conclude, that the MPT fragment of the FA remains largely intact following HT treatment of the FA and is responsible for the widely reported increase in the fluorescence quantum yield. Absorption and Raman data provide further insights into the chemical state of the MPT fragment which is likely to be stabilised by the OH groups present in the aqueous synthesis environment. The UV–VIS absorption (Fig. 1) and the Raman (Fig. 4) data suggest presence of 6-formylpterin^20–22^, while formation of the pterin-6-carboxilic acid also cannot be entirely excluded^20^. It has been reported^4,6,23,24^ that HT-treated FA retains its folate receptor targeting properties which, together with the results of this study, strongly suggest that the MPT shape and the key surface groups responsible for the folate receptor binding remain intact (at least up to 120 min at 200 °C) since FA binding to the receptor involves both shape and charge complementarity^7^. At this point we should point out that an increase in fluorescence emission has been well documented in case of exposure of FA to the UV light and the exact mechanism of the FA breakdown has been investigated on the molecular scale using DFT calculations^13^. However, unlike in the case of the UV exposure that results in electronic excitation, HT processing of FA doesn’t lend itself to the DFT treatment. Thus, although the exact FA breakdown pathway under HT treatment is not clear, we can conclude that he most likely fluorescent product of the breakdown is 6-formylpterin (see Fig. S2).

## Conclusions

Our findings show that the result of HT treatment of the FA for 120 min at 200 °C is breakdown of the acid into fragments. Comparison of experimental and DFT-calculated UV–VIS and Raman data (see Figs. 2 and 4) suggest that only peaks associated with glutamate and PABA have disappeared as a result of HT treatment. It further follows from the analysis of the UV–VIS absorption, fluorescence emission, IR and Raman data that the MPT responsible for the light emission remains largely intact. We concluded that the excitation-independent blue light emission widely reported for carbon nanoparticles prepared from FA is of molecular nature and is due to enhanced emission from the MPT following removal of the FA fragments responsible for the fluorescence quenching (i.e. PABA). Our experimental findings are supported by the DFT calculations which allow for atomic-scale investigation of the evolution of FA under HT treatment.

HT treatment has been widely used to produce what is frequently described as carbon nanoparticles. However, in many cases^12,15^ it is some sort of molecular species that can be responsible for the light emission both on the account of the reported high quantum yields and PL emission range (typically blue). Here we demonstrated that it is certainly the case for HT-treated FA. At the same time, insights gained in this work could be applied to a variety of molecules to alter their emission and/or to produce new fluorophores utilising reduced stability of molecular fragments (e.g. PABA and Glu). This should be particularly true for molecules consisting of aromatic rings and non-conjugated chains. Finally, analysis we carried out indicates that the DFT-based screening can provide a pathway to novel fluorophore development using HT synthesis route.

## Methods

### Hydrothermal treatment of folic acid

Hydrothermal treatment was carried out according to the protocol described in our recent publication^22^. The typical procedure includes preparation of FA water suspension (1000 ml), using 0.44 g (or 0.0018 g) of FA, allowing to obtain solution 1 × 10^–3^ M (or 4 × 10^–6^ M). A portion of this suspension (3 ml) was then transferred into a glass cup, placed into the Teflon cup with tight-fitting cover, put inside stainless steel autoclave and heated at 200 °C for a certain selected time. The resulting solution was cooled at room temperature and centrifuged (20 min, 7500*g*). After centrifugation solutions were stored at 4 °C.

### Sample characterisation

Luminescent and excitation spectra were measured with a spectrofluorimeter Cary Eclipse (Agilent Technologies, Australia). Absorbance spectra of solutions were recorded using Shimadzu UV-1800 UV/Visible Scanning Spectrophotometer (Shimadzu, Japan**)** in a standard 10-mm quartz cuvette. To exclude possible self-precipitation due to exposure to UV-irradiation FA colloids of 10^–4^ M and 10^–3^ M concentration were diluted to 10^–5^ M immediately before spectra recording. The relative quantum yields (QY) of samples were calculated using quinine sulfate as a reference.

Raman spectra were acquired with a Renishaw inVia Raman (Renishaw, UK) and Horiba LabRam HR (Horiba, France) spectrometer sunder 532 nm irradiation (200 μW, power at 0, 1%) and. The integration time was 30 s per spectrum. A drop of samples diluted to 10^–4^ M was deposited on a glass slide for each measurement.

FT-IR spectrometer Nicolet 6700 (Thermo Scientific, USA) was used to record infrared spectra. Samples were dried in advance and prepared in an inert atmosphere in an amount of 3 mg. Preparation of solid samples for registration of the infrared spectra was carried out in the pressing of tablets with alkali metal halides (KBr), at room temperature.

### DFT calculations and spectra modelling

Molecular models for all structures have been obtained from the PubChem—an open chemistry database at the National Institutes of Health, USA. All quantum chemical calculations were performed using Gaussian 09 suite of programs^25^ Geometry optimization and frequency calculations (including Raman intensities) of studied molecules have been achieved using the hybrid Density Functional Theory approach B3LYP^26,27^ with Pople type double ζ basis sets augmented by polarization functions on all atoms (namely 6-31G(*d*,*p*))^28,29^. In order to calculate electronic excitation wavelengths (λ_TD-DFT_) and the associated oscillator strength parameters (f_TD-DFT_), TD-B3LYP calculations have been performed using 6-31G(*d,p*) basis sets. For geometry optimizations and spectroscopic parameters computations, the SMD model^30^ has been employed to simulate bulk solvent effects (i.e. water; ε = 78.3553 and ε(inf) = 1.806874). In order to compare theoretical and experimental data, Raman and UV–Visible spectra have been simulated by performing convolution with Gaussian type functions. For Raman spectra, convolution has been performed thanks to the Molden program^31,32^ using 20.0 cm^-1^ width at half-length. For electronic transition spectra, convolution of each calculated transition and the sum of these Gaussian functions on the whole wavelength range have been achieved using the following equation:

$$I\left( {\lambda_{Spec} } \right) = \mathop \sum \limits_{i = 1}^{NStates = 30} f_{TD - DFT}^{i} e^{{ - \frac{{\left( {\lambda_{Spec} - \lambda_{TD - DFT}^{i} } \right)^{2} }}{BW}}}$$

where $I\left( {\lambda_{Spec} } \right)$ is the intensity of the simulated spectrum at $\lambda_{Spec}$, $\lambda_{TD - DFT}^{i}$ and $f_{TD - DFT}^{i}$ are the wavelength and the oscillator strength of the *i*th TD-DFT excited state, respectively. The parameter BW was arbitrary set to 300 nm^2^ in order to achieve a fixed width at half-length close to 30 nm. No correction (red or blue shift) has been applied on the theoretical wavelengths when simulating whole UV–Visible spectra. Similarly, no correction parameter has been applied to the calculated frequencies for the simulation of Raman spectra.

## Supplementary information

Supplementary Information.
