# Supplementary material for: Molecular nature of breakdown of the folic acid under hydrothermal treatment: a combined experimental and DFT study
Source: Sci Rep. 2020 Nov 12;10:19668. doi: 10.1038/s41598-020-76311-y (PMC7661697; doi:10.1038/s41598-020-76311-y)
Supplement: Supplementary file 1 — Supplementary Information. [file 41598_2020_76311_MOESM1_ESM.docx]

Supporting Information

Molecular nature of breakdown of the folic acid under hydrothermal treatment: a combined experimental and DFT study

Anna M. Abramova, Alina A. Kokorina, Olga A. Sindeeva, Franck Jolibois, Pascal Puech, Gleb B. Sukhorukov, Irina Y. Goryacheva, Andrei V. Sapelkin

**Table S1**. Fluorescence quantum yield data for the folic acid before and after HT treatment.

| Quantum Yield,% | | | | |
| --- | --- | --- | --- | --- |
| FA concentration, М | Hydrothermal Treatment Time, min | | | |
|  | 0 | 60 | 120 | 180 |
| 1*10^-4^ | 0,4 | 9 | 9,5 | 9 |
| 1*10^-3^ | 0,3 | 8,5 | 10 | 6 |


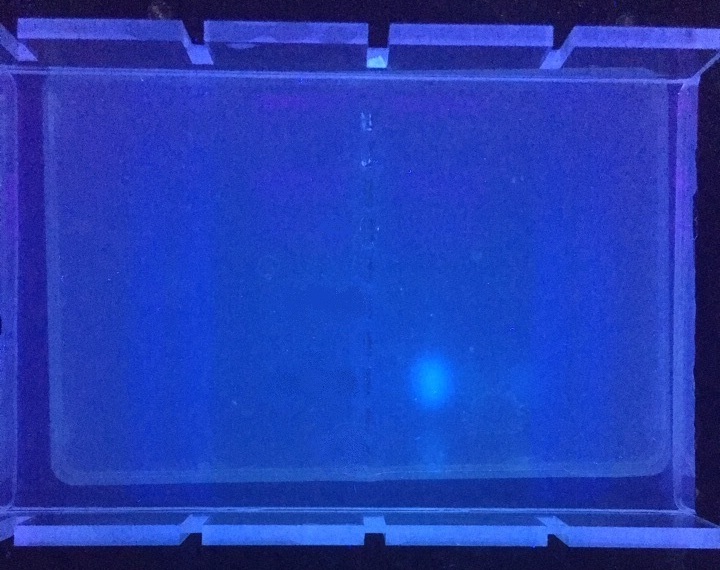


**Figure S1.** Gel-electrophoresis image of the folic acid sample before and after high temperature treatment.


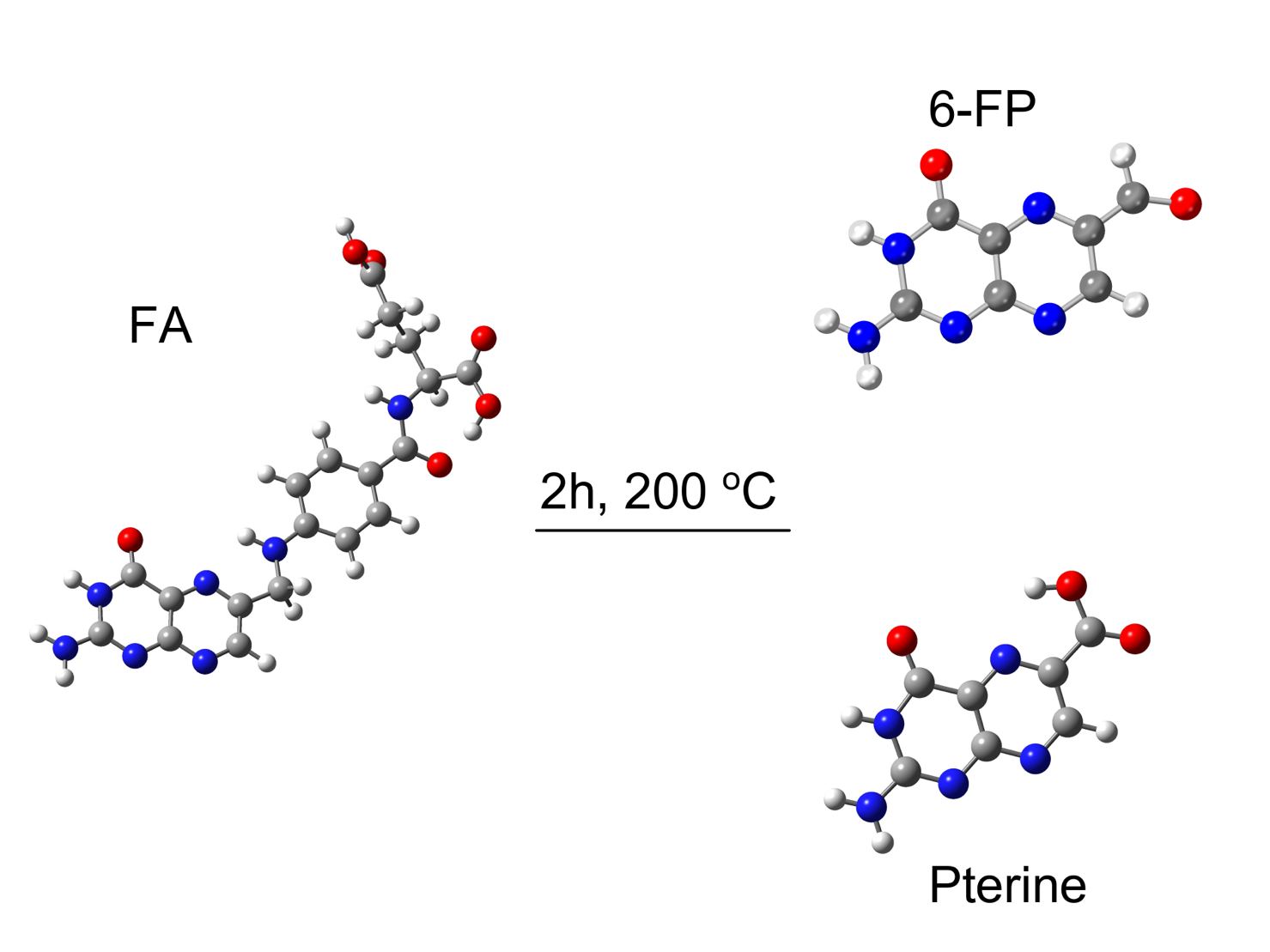


N

O

C

H

**Figure S2.**  Molecular structure of folic acid (FA) and possible molecular species formed as a result of the hydrothermal treatment: pterine-6-carboxylic acid (pterine) and 6-formylpterin (6-FP).
